# The Association Between Physical Activity and General Life Satisfaction in Lower Secondary School Students: The Role of Individual and Family Factors

**DOI:** 10.1007/s10597-018-0309-x

**Published:** 2018-08-03

**Authors:** Dorota Kleszczewska, Anna Dzielska, Ferdinand Salonna, Joanna Mazur

**Affiliations:** 1 Institute of Mother and Child Foundation, ul. Kasprzaka 17a, 01-211 Warsaw, Poland; 2 Department of Child and Adolescent Health, The Institute of Mother and Child, Warsaw, Poland; 3 Palacký University of Olomouc, Olomouc, Czech Republic

**Keywords:** Adolescents, Life satisfaction, Physical activity, Family affluence, Self-esteem

## Abstract

The objective of the paper was to investigate the association between PA and general life satisfaction in adolescents, taking into account family affluence and selected psychological, family and school factors. The survey (2015) involved 4085 Polish lower-secondary school students. Life satisfaction was measured with the abridged Students’ Life Satisfaction Scale (SLSS). Vigorous Physical Activity, self-esteem, family affluence, family relations and the perception of the school environment were considered as independent variables. Hierarchical and path models were applied. The mean SLSS score was M = 4.66 (SD = 2.23), and 36.9% of its variability was explained—mainly by self-esteem. The impact of PA on self-esteem became stronger when family affluence decreased, which led to the conclusion that material status may modify the impact of behavioural factors on the SLSS scores’ variability in adolescence.

## Introduction

Interest in the effect of physical activity on health is considerable, but for years the focus has been mainly on its significance for preventing such diseases as obesity (Sybilski et al. 2007), cardiovascular disease (Kruk and Aboul-Enein 2006), diabetes type 2 (De Backer and De Bacquer 2004) and cancer (Laaksonen et al. 2005). Recently, there has been increasing discussion about the association between physical activity and psychosocial health. A systematic review of 30 selected papers published by Eime et al. (2013), suggests that physical activity may provide protection against psychosocial health problems such as depression (Hughes et al. 2013), social anxiety, low self-esteem and even against suicidal thoughts.

The World Health Organization (WHO) strategy relating to physical activity for the European region for 2016–2025, published in September 2015, included physical activity as a necessary condition for both physical and mental health. The strategy refers to all groups: children, adolescents, adults and senior citizens (WHO 2015). It is recommended that children and adolescents engage in moderate, aerobic activity (every day for a minimum of 60 min) as well as in vigorous activity several times a week, including bone- and muscle-strengthening exercises.

In research on the health benefits of physical activity in young people, the effect of demographic factors, such as gender, age and place of residence, is taken into account (Abd-Latifa et al. 2012). Few studies have considered the impact of family affluence (Witana and Szpak 2009). This indicator and its relationship with the physical activity of adolescents has been described in the literature in the context of its impact on the self-esteem of young people (McPhie and Rawana 2012).

In the findings of the most recent round of the international Health Behaviour in School-aged Children (HBSC) survey, it can be observed that children from more affluent families, from almost all countries, are more satisfied with their lives and they are also more physically active. Poland has an average position in international rankings in terms of physical activity, and is much less favourable in terms of life satisfaction. In Poland, in comparison with other countries, increasing social inequalities in these two indicators have been observed (Inchley et al. 2016). It was thus concluded that more extensive analysis should be conducted into the association between life satisfaction, physical activity and family affluence.

Life satisfaction has been regarded as a reliable indicator of personal well-being and psychological development in adolescence. An assumption was made that the fundamental determinants of general life satisfaction in adolescents include self-satisfaction, family satisfaction and school satisfaction (Mazur et al. 2016; Strózik et al. 2016).

General life satisfaction may be treated as a positive mental health indicator, contrary to many more frequently considered negative indicators (Moulin et al. 2017). Repeated research has shown a socioeconomic gradient in subjective health related outcomes in adolescence (Schutte et al. 2014), however only a few studies have investigated the interaction between behavioural factors and family socioeconomic position as predictors of general well-being (Bannink et al. 2016). This research attempts to explain the mechanisms of how physical activity affects general life satisfaction, including problems which are specific to various social groups.

### Objective of the Study

The objective of the study was to investigate the association between physical activity and general life satisfaction in lower secondary school students, taking into account the material status of their families and other determinants of life satisfaction: self-esteem, family relations, school climate and school achievements.

A hypothesis was adopted that family affluence modifies the association between physical activity and life satisfaction in adolescents, affecting a range of direct and indirect relationships (Elgar et al. 2015).

The following research questions were raised:

- Does physical activity remain a significant predictor of life satisfaction in adolescents, adjusting for the impact of other potential sociodemographic factors like gender and age?
- Is the association between physical activity and life satisfaction direct or indirect in nature? Which factors can be considered mediators of this association?
- Does the family affluence affect the strength of the association between physical activity and life satisfaction?

## Materials and Methods

The study comprised 4085 students (48% boys and 52% girls) from 70 lower secondary schools from across Poland. The “Health and School 2015” survey was conducted in the second semester of the 2014/2015 school year as part of a research project of the National Science Centre, implemented by the Institute of Mother and Child in Warsaw. It was an anonymous, mostly computer-assisted web survey. The directors of the randomly sampled schools were informed about the aim of survey as well as their supervisory units. External trained interviewers were responsible for data collection, in some cases with school pedagogue help. Consent signed by the parent was obtained before youth entered the survey. The questionnaire and the survey organization schema has been accepted by Bioethical Commission operating at the Institute of Mother and Child. The students had a right to refuse answering questions too sensitive for them. In each school one class per grade was randomly selected (210 classes in total). The project was carried out by the same research center that coordinates HBSC study in Poland, covering similar age group (in HBSC 11, 13 and 15 years olds). The results of Health and School 2015 and HBSC 2014 study were compared by selected demographic characteristics as well as a number of health and school perception indicators. The large consistency of results indicates the representativeness of the sample used in the current project (Mazur et al. 2015).

The response rate was 84.8%. The respondents were residents of big cities (with populations of over 100,000), small towns and rural regions (21.1; 33.7 and 45.2%). The survey comprised the 1st, 2nd and 3rd grades of lower secondary schools (33.1; 35.6; 31.3%). The average respondent age was 14.91 years; the age range was 13–17; however, only 0.9% of the respondents were above the age of 16.5 years (Mazur et al. 2015).

Responses relating to the following areas were answered and analysed.

### Life Satisfaction

The shorter version of the Huebner scale [The Students’ Life Satisfaction Scale (SLSS)] was used as a dependent variable. It is a tool used to assess life satisfaction in studies of young people aged 8–18 (Huebner et al. 2003), which provides an alternative to the Cantril ladder used in the HBSC survey. A short version of this scale was used. Students were asked three questions such as: last month how often you felt: *(1) You are satisfied with the course of your life; (2) You have a satisfying life; (3) Everything that happens to you is good*. There were four categories of answers: *never, sometimes, often, almost never*; the scores were 0–3. The summary index of life satisfaction ranged between 0 and 9 points.

### Family Affluence

Family affluence was measured with a modified, 6-item version of the Family Affluence Scale (FAS) developed by HBSC network. The questions referred to: having one’s own room, the number of cars in the family, the number of computers in the family, going on holidays with the family, the number of bathrooms in the home and whether it was fitted with a dishwasher. The range for this scale was 0–13 points. Further analyses made use of the family affluence indicator in the form of a continuous variable: families were divided into poor, average and affluent (Torsheim et al. 2016).

### Physical Activity

*Vigorous Physical Activity (VPA)* was defined by the WHO as requiring a significant effort, making breathing quicker and the pulse faster, equal to MET ≥ 6 (metabolic equivalent) compared to a person seated at rest. The WHO provides examples of the following activities categorized as VPA: running; fast cycling; fast swimming; team sports, such as football or basketball (WHO 2004).

The VPA level was based on one question—*How often, in your free time, out of your school classes, do you engage in physical exercise during which your physical effort is great, i.e. you feel that you are short of breath and sweating?*—with seven categories of answers. The VPA indicator was used in the analyses as a quasi-continuous variable (a range of 0–6 points) divided into five categories: *rarely or never* (three most negative responses combined); *once a week; 2–3 times a week; 4–6 times a week; every day*.

### Other Determinants of Life Satisfaction

*The self-esteem scale* originates in the Child Health and Illness Profile-Adolescent Edition questionnaire (CHIP-AE; Starfield et al. 1995). Young people selected from a four-point scale how much they agree with three statements (e.g. I like myself just the way I am). The scale has a single-factor structure and a reliability of 0.853 (Cronbach’s alpha). It has a scale from 0 to 9, where a high score indicates higher self-esteem.

*The scale of the perception of family relations* is also taken from the CHIP-AE questionnaire. Young people described how often in the last 4 weeks they experienced positive feelings in family relations (e.g. You were happy to be a member of your family). The scale has a single-factor structure and a reliability of 0.848 (Cronbach’s alpha). It has a scale from 0 to 12, where a high score indicates good relations in the family.

When analysing the impact of the school environment, *the assessment of school performance* in comparison with other students in the class (on a visual 0–10 point scale) was taken into consideration along with a general perception of the school environment (school climate). *The scale of the school climate* taken from the HBSC research protocol is composed of four questions relating to support from other students and a feeling of belonging to the school (e.g. I like being at school). Five categories of answers were offered (from *I definitely disagree*, to *I definitely agree*). The summary scale has a single-factor structure and a reliability of 0.750 (Cronbach’s alpha). It has a range of 0–16 points where a high score indicates a positive assessment of the school environment.

### Data Analysis Methods

The following statistical methods were used in the following order:

- In order to examine the psychometric qualities of the SLSS scale, an exploratory factor analysis (principal component analysis) was conducted along with an analysis of scale reliability using Cronbach’s alpha.
- The normality distribution of the dependent variable was investigated using the Kolmogorov–Smirnov test and whether the skewness differs significantly from zero.
- Using one-factor analysis of variance (ANOVA), mean values for life satisfaction were compared according to sociodemographic groups and the VPA level.
- The association between life satisfaction and potential determining factors were investigated.
- Hierarchical linear regression models were estimated, where the SLSS scale was the dependent variable. In the first step, gender, age, family affluence and physical activity level were all included in the model. In the second step, a family relations assessment was added, and in the third step, school-related factors were added. The last step consisted of adding self-esteem. The models were compared with the change of the R^2^ factor. The variables that were the main SLSS’s predictors in the classic procedure of stepwise variable selection were also checked. The strength of the association between physical activity and general life satisfaction was analysed in the social groups; simple path models were estimated; Sobel’s mediation test was used.

The data was analysed using IBM SPSS v.21 (PS Imago) and AMOS 21.

## Results

### Life Satisfaction

The results of the analyses conducted suggest high reliability (Cronbach’s alpha = 0.841) of the SLSS scale—the short Huebner’s scale. In the course of the exploratory factor analysis (principal component analysis), one factor was singled out that explains 75.9% of common variance. The principal component loadings for items included in the scale ranged from 0.846 to 0.896. The distribution of the life satisfaction index—with this sample size—deviated from the normal one according to the K–S test (p < .001); however, it was symmetrical. Skewness was 0.072 (standard error 0.039), which suggests that skewness is not significantly different from zero. As a result, it was concluded that the use of methods assuming normal distribution was permissible.

Table 1 shows the mean (M) values of the life satisfaction index according to sociodemographic factors (gender, age, family affluence) and VPA in the respondents’ free time.

**Table 1:** Life satisfaction in lower secondary school students according to the sociodemographic factors and physical activity (mean values and standard deviation)

Independent variable	N	%	Mean	SD	p^a^
Gender					
Boys	1907	47.6	4.86	2.20	< .001
Girls	2102	52.4	4.47	2.25	
Class of lower secondary school					
I	1319	32.9	4.78	2.21	< .05
II	1428	35.6	4.56	2.22	
III	1262	31.5	4.64	2.27	
Family affluence (FAS)					
Poor (0–5)	946	24.5	4.27	2.18	< .001
Average (6–9)	2177	56.4	4.70	2.22	
Affluent (10–13)	740	19.2	4.99	2.26	
Physical activity (VPA)					
Rarely or never	545	13.7	4.23	2.26	< .001
1 time per week	512	12.8	4.32	2.11	
2–3 times per week	1160	29.1	4.67	2.13	
4–6 times per week	834	20.9	4.84	2.14	
Everyday	935	23.5	4.90	2.42	

^a^ANOVA test

On average, life satisfaction among the respondents was M = 4.66 (SD = 2.23), which is 52% of the maximum possible score. The mean Huebner index was significantly higher in girls than in boys (p < .001). Age contributed less to the differences in life satisfaction (p < .05). This was a curvilinear correlation; the lowest level of life satisfaction was found in second-graders. Statistically significant differences were also found in relation to family affluence (p < .001) in favour of young people from more affluent families (an increase of the mean by 0.72). The index of life satisfaction according to the SLSS scale, increased with the time devoted to physical activity.

### A Simple Analysis of the Correlations Among the Investigated Variables

Table 2 shows a simple analysis of the correlations among general life satisfaction measured with the SLSS scale and potential determining factors, as well as mutual correlations among those factors. General life satisfaction indicates a significant correlation with six analysed factors: the strongest with self-esteem and with quality of family relations. Among analysed variables only perception of the school atmosphere did not show a significant correlation with family affluence. Physical activity also has a weak correlation with the school-related variables but the strongest with self-esteem.

**Table 2:** Correlation matrix

		1	2	3	4	5	6	7
		Significance level (p)						
1. Life satisfaction	Spearman correlation		.000	.000	.000	.000	.000	.000
2. Family affluence		.119		.000	.000	.447	.000	.000
3 Self-esteem		.527	.075		.000	.000	.000	.000
4. Family relationships		.414	.124	.332		.000	.000	.000
5. School climate		.239	− .012	.188	.172		.000	.134
6. Academic achievements		.154	.149	.158	.141	.085		.565
7. Physical activity		.110	.073	.183	.058	.024	.009	

### Multi-factor Determinants of General Life Satisfaction

**Fig. 1:**
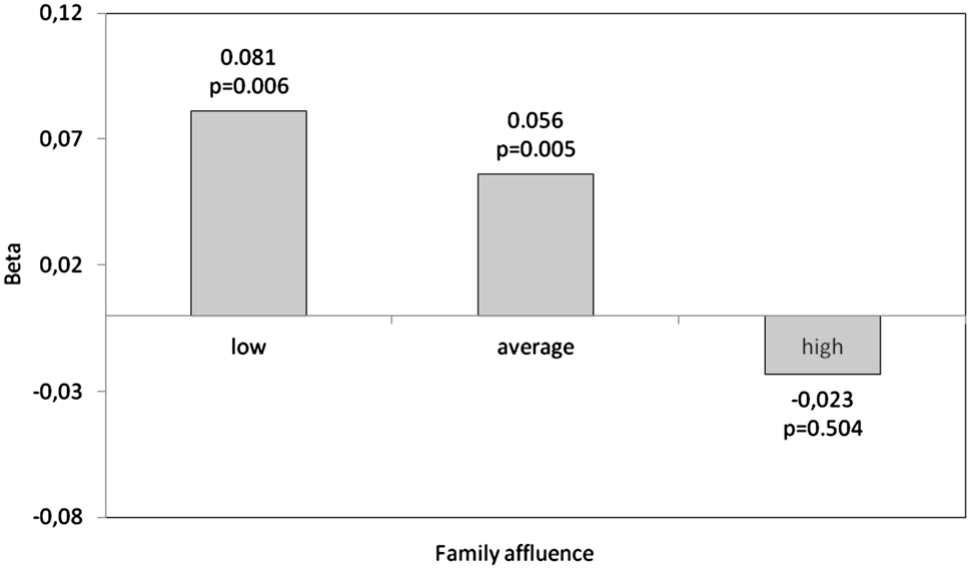
Standardised regression coefficient (beta) of life satisfaction in relation to physical activity according to family affluence. Models adjusted for age, gender, self-esteem, family relations, academic achievements and school climate

Table 3 shows four models which identify the variability of the general SLSS index. The first model included only demographic factors (gender, age), family affluence and physical activity. It explains only 2.8% of the variability in life satisfaction; all factors (except age) are significant. Adding family relations does significantly improve the fit of the model (Model 2), while variables which describe the school (Model 3) do not result in such a significant improvement. In Models 2 and 3, the inference related to the impact of sociodemographic factors and physical activity does not change but VPA continues to be a significant predictor. Only Model 4, which takes into account self-esteem among the independent variables, differs significantly from the previous models. The indicator of the goodness of fit increases by another 14.2%. An important effect of age manifests itself, and the impact of gender and physical activity decreases. It can therefore be assumed that self-esteem “replaces” physical activity in the model. A new estimate of Model 4, carried out using the method of stepwise variable selection, indicates that self-esteem is the most important factor determining general life satisfaction in adolescents. Analogical models, estimated for three groups of affluence, indicate that in Model 3 physical activity remains a significant SLSS predictor only in poor and average families, while in the most affluent families its impact is insignificant (Fig. 1). Regardless of family affluence, however, once self-esteem is introduced to the model, the impact of physical activity is visibly reduced.

**Table 3:** Results of hierarchical linear regression

Independent variables	Model 1		Model 2		Model 3		Model 4	
	â	p	â	p	â	p	â	p
Age	− 0.015	.370	0.003	.829	0.013	.381	0.039	.004
Gender	0.076	.000	0.067	.000	0.082	.000	− 0.014	.305
Family affluence (FAS)	0.109	.000	0.062	.000	0.053	.000	0.049	.000
Physical activity (VPA)	0.081	.000	0.056	.000	0.045	.002	0.004	.745
Family relationship			0.404	.000	0.358	.000	0.237	.000
School climate					0.170	.000	0.105	.000
Academic achievements					0.099	.000	0.045	.001
Self-esteem							0.429	.000
R^2^	0.028		0.188		0.227		0.369	

â—Standardized regression coefficient

### An Analysis of the Effect of Mediation

**Fig. 2:**
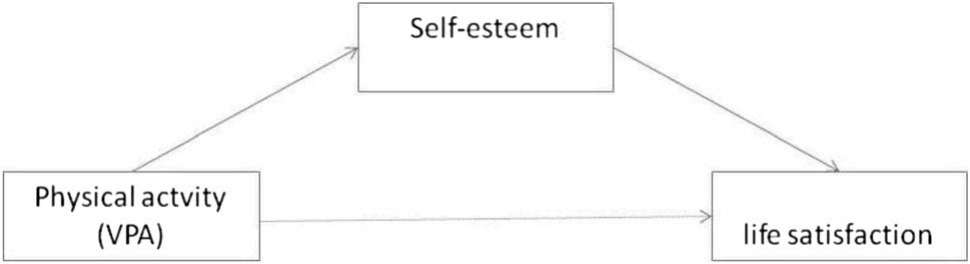
Self-esteem as mediator of the relationship between VPA and life satisfaction

Based on the results of previous analyses shown in Tables 2 and 3, the hypothesis was drawn that self-esteem might be the mediator of the relation between physical activity and general life satisfaction in adolescents in accordance with the simple path model shown in Fig. 2. The assumption was confirmed by conducted analysis and corroborated by Sobel’s mediation test. Table 4 also shows the results of the estimation of the path model in three groups of affluence. An important effect of the mediation was manifest in all groups; the direct correlation between physical activity and SLSS is also insignificant in all of them. However, it should be noted that the coefficients of regression show that the effect of VPA on SLSS systematically increases as the family affluence of the surveyed adolescents becomes worse.

**Table 4:** Results of simple path model according to family affluence

	Total group	Low FAS	Average FAS	High FAS
Standardized regression weights (p)				
VPA → self-esteem	0.160 (< .001)	0.174 (< .001)	0.155 (< .001)	0.125 (< .001)
Self-esteem → SLSS	0.532 (< .001)	0.557 (< .001)	0.534 (< .001)	0.505 (< .001)
VPA → SLSS	0.017 (.205)	0.027 (.319)	0.010 (.600)	− 0.017 (.599)
Mediation effect^a^	9.85	5.27	5.80	5.27

^a^Self-esteem mediates the relationship VPA → SLSS (Fig. 1); Sobel test; significant at p < .001 in all cases

## Discussion

The aim of the paper was to confirm the relationship between physical activity and general life satisfaction in lower secondary school students, while taking into account the socio-economic status of their families and other potential determinants.

This study concludes that physical activity remains a significant predictor of life satisfaction in adolescence. However, it has been revealed that self-esteem is a more important predictor of life satisfaction and it modifies the relationship between PA and SLSS.

The link between physical activity and mental health is strongly proven in the literature. Physical activity becomes a factor in reducing anxiety and stress, and has a positive effect on curing depression. Biddle and Mutrie (2008) also indicated the effect of physical activity on self-esteem, self-perceptions, mood and psychological well-being, selected areas of cognitive function and psychological adjustment. Furthermore, the emphasis is on the role of self-esteem and satisfaction with life, also referring to physical activity. For example, under the Kipp’s (2016) survey on US adolescence, it was demonstrated that high self-esteem determines life satisfaction in adolescents, and that physical activity has a significant impact upon it. The positive impact of physical activity on self-perception may be considered as a benefit of being active in adolescence (Stein et al. 2007). Numerous international studies identify a link between the self-esteem of adolescents and their general life satisfaction (Ahmed et al. 2016). Both of these indicators of mental health may be analysed in the context of family affluence. In a well-known UK Millennium Cohort Study, a strong correlation between family affluence and the mental health of 11-year-olds born at the beginning of this century was demonstrated (Bannink et al. 2016). Our analysis may be treated as an extension of this kind of research, but according to an older age group and from an Eastern European perspective. Firstly, we have assumed that self-esteem is a direct determinant of life satisfaction (it is not just an alternative indicator of mental health). Then, physical activity, which can be stimulated in intervention studies, was introduced to the estimated models.

Physical activity, family affluence and gender were all found to be factors affecting the variability in life satisfaction among young people. The effect of age was weaker but the group was reasonably homogeneous in this respect. A positive relationship between family socioeconomic status and adolescent life satisfaction was also noted in research conducted among young Italians (Lazzeri et al. 2014), and in the results of analyses relating to 35 countries participating in the 2006 HBSC survey (Levin et al. 2011). In the study of Zullig and White (2011), engagement in group sports was a factor that led to increasing feelings of life satisfaction in both genders. Avoiding VPA throughout the week, negatively affected the level of life satisfaction in girls. Similar conclusions were drawn in a study by Schmalz et al. (2007). These results show that physical activity is conducive to positive self-esteem, especially among younger adolescents. The Youth Risk Behaviour Survey conducted by Valois et al. (2004) in South Carolina, demonstrated mutual dependence—PA strengthens adolescents’ life satisfaction, but those young people who were less satisfied with their lives were also less physically active. The mutual nature of this correlation should be emphasized at this point. Life satisfaction and generally positive attitudes towards oneself tend to increase among people who are physically active, but at the same time it motivates them to continue a healthy lifestyle. PA is positively associated with self-perceived dispositional resilience among those with high trait anxiety. As such, for those young people who are at risk of mental health problems, PA may facilitate resilience and reduce the likelihood of developing stress-related symptoms or disorders (Hegberg and Tone 2015).

In the study discussed above, there is an attempt to answer the question of whether the intensity of the association between physical activity and life satisfaction changes in groups of varying affluence. The positive impact of physical activity on life satisfaction was most visible among adolescents from low income families; no such correlation was observed in the most affluent group of young people.

It should be emphasized that the indicator of physical activity used in the analyses was the question about vigorous physical activity undertaken in the respondents’ free time, excluding physical education classes at school. The percentage of young people who had almost no PA, was twice as high in low income families in comparison to affluent ones. The probable cause of this situation may be explained by the limited availability of various forms of extracurricular physical activity, especially this extra fee-paying ones. Many papers have pointed out that the participation of young people from disadvantaged backgrounds in various extracurricular classes may prevent social exclusion. It may also fulfil many psychological needs such as: increasing feelings of belonging, experience of support and care, building faith in one’s abilities, and others. Benefits from free-time activities can alleviate the deficiencies caused by growing up in a less affluent environment (Kipp 2016; Nihill et al. 2013).

It is worth noting that young people from families with lower material status have fewer opportunities to practice sports and, generally, to be physically active compared to their more affluent peers (Scheerder et al. 2005; Holt et al. 2011). In this context, it is important for decision makers to create a friendly environment for practicing sports, and to propose a wide range of free-of-charge sporting activities for young people from low income families.

The results are also very significant in light of the discussion about health inequalities among the population of Poland (WHO 2012). Children and young people have an important place in this discussion because their current health is going to affect their future health (Kakinami et al. 2013). The study proves the utility of analysing health determinants in social groups; it also indicates the need to carry out a combined assessment of the effect of sociodemographic and behavioural factors. An added value is noting that simple analyses of the findings of cross-sectional studies may help determine the directions taken by intervention measures.

### Limitations of the Study

The findings in the present study need to be interpreted in the light of some methodological limitations. First, due to cross-sectional design, causation cannot be inferred. Second, in future more determinants of adolescent well-being should be included. The advantage of the present study is the use of a large representative sample of adolescents. In the light of the available knowledge this is one of the few studies that explores the complex effects of interaction, including social determinants. This means that the relationship between physical activity and mental health of adolescents is analyzed both in the context of modifying and moderating factors.

## Conclusions

1. Young people’s self-esteem—which is the main predictor of general life satisfaction in adolescents—increases along with their level of physical activity.
2. The positive impact of physical activity on life satisfaction is more visible among less affluent adolescents.
3. It is important to create a sport-friendly environment, and to engage young people from the poorest families in physical activity during their free-time through broadening access to free sports classes, sports scholarships and other forms of assistance. This is also an important method of enhancing adolescent life satisfaction and enhancing social inclusion.
